# Protective effect of rutin supplementation against cisplatin-induced Nephrotoxicity in rats

**DOI:** 10.1186/s12882-017-0601-y

**Published:** 2017-06-15

**Authors:** Ali R. Alhoshani, Mohamed M. Hafez, Sufia Husain, Abdel Malek Al-sheikh, Moureq R. Alotaibi, Salim S. Al Rejaie, Musaad A. Alshammari, Mashal M. Almutairi, Othman A. Al-Shabanah

**Affiliations:** 1 Department of Pharmacology and Toxicology, College of Pharmacy, King Saud University, P.O. Box 2457, Riyadh, 11451 Kingdom of Saudi Arabia; 2 Department of Pathology, College of Medicine, King Saud University, P.O. Box 2457, Riyadh, 11451 Kingdom of Saudi Arabia

**Keywords:** Cisplatin, Nephrotoxicity, P38 MAPK, Gene expression

## Abstract

**Background:**

Cisplatin (CP) is commonly used in the treatment of different types of cancer but nephrotoxicity has been a major limiting factor. Therefore, the present study aimed to study the possible protective effect of rutin against nephrotoxicity induced by cisplatin in rats.

**Methods:**

Forty male Wistar albino rats were randomly divided into 4 groups. Rats of group 1 control group intraperitoneal (i.p.) received 2.5 ml/kg, group 2 CP group received single dose 5 mg/kg cisplatin i.p. group 3 rutin group orally received 30 mg/kg rutin group 4 (CP plus rutin) received CP and rutin as in group 2 and 3. Kidneys were harvested for histopathology and for the study the gene expression of c-Jun N-terminal kinases (*JNK*), Mitogen-activated protein kinase 4 (*MKK4*), *MKK7*, P38 mitogen-activated protein kinases (*P38*), tumor necrosis factors alpha (*TNF-α*), TNF Receptor-Associated Factor 2 (*TRAF2*), and interleukin-1 alpha (*IL-1-α*).

**Results:**

The cisplatin single dose administration to rats induced nephrotoxicity associated with a significant increase in blood urea nitrogen (BUN) and serum creatinine and significantly increase Malondialdehyde (MDA) in kidney tissues by 230 ± 5.5 nmol/g compared to control group. The animal treated with cisplatin showed a significant increase in the expression levels of the *IL-1α* (260%), *TRFA2* (491%), *P38* (410%), *MKK4* (263%), *MKK7* (412%), *JNK* (680%) and *TNF-α* (300%) genes compared to control group. Additionally, histopathological examination showed that cisplatin-induced interstitial congestion, focal mononuclear cell inflammatory, cell infiltrate, acute tubular injury with reactive atypia and apoptotic cells. Rutin administration attenuated cisplatin-induced alteration in gene expression and structural and functional changes in the kidney. Additionally, histopathological examination of kidney tissues confirmed gene expression data.

**Conclusion:**

The present study suggested that the anti-oxidant and anti-inflammatory effect of rutin may prevent CP-induced nephrotoxicity via decreasing the oxidative stress, inhibiting the interconnected *ROS/JNK/TNF/P38* MAPK signaling pathways, and repairing the histopathological changes against cisplatin administration.

## Background

Cisplatin (CP) is a chemotherapy commonly used in cancer treatment including head, neck, ovarian, and testicular cancers [1, 2] but is associated with nephrotoxicity in 28–36% of patients receiving an initial dose (50–100 mg/m^2^) of cisplatin [3]. The accumulation of high concentrations of cisplatin in the kidneys caused nephrotoxicity [4]. This serious complication is contributed to limiting its clinical use. The intermission of cisplatin remains the only choice in the case of progressive renal failure [5]. Cisplatin-induced nephrotoxicity through apoptosis and necrosis [6], vascular factors [7], and inflammation of the tubules [8]. The development of renal tubule injury is caused by the oxidative stress induced by cisplatin [9–12]. The reactive oxygen species (ROS) and reactive nitrogen species (RNS) production [13] alter the structure and function of cellular membranes [14]. In addition to their accumulation in kidney and lysosomes [15] explained the mechanisms for CP-induced acute nephropathy [13]. Although numerous mechanisms for CP-induced nephrotoxicity such as mitochondrial dysfunction, inflammation, DNA damage, oxidative stress and apoptosis had been studied, the precise mechanism is not well understood [16, 17]. Therefore, the free radical scavengers and the antioxidants agent can prevent cisplatin-induced nephrotoxicity.

Cisplatin damages the DNA resulting in apoptosis induction [18]. In response to cisplatin, several signaling pathways, which can be activated by lipid peroxidation and oxidative stress, modulate the cell survival or apoptosis [18, 19]. The mitogen-activated protein kinase (*MAPK*) pathways regulate differentiation, proliferation, apoptosis and are activated by chemical and physical stresses [20]. The three major *MAPK* pathways terminate in *ERK*, *p38*, and *JNK/SAPK* enzymes. Cisplatin is known to activate these three pathways in various cell lines including renal epithelial cells [21, 22]. *p38 MAPK* was involved in inflammation, cell cycle regulation, and differentiation [23] but its role in cancer therapy is not clear. Recently, some investigator suggests that *p38 MAPK* is able to control the p53-mediated response to cisplatin [24].

The interleukin-1 *(IL-1*) made up of 11 proteins encoded by 11 different genes [25] and its main function, in response to tissue injury or damage, is to control the pro-inflammatory reactions [26]. Activation of *IL-1* lead to activation of some genes such as Mitogen-activated protein kinase kinase 4 (*MKK4*) and (*MKK7*) which activate *JNK* [27, 28], and *MKK4*, *MKK3*, and *MKK6* activate *p38 MAPK* [29].

Flavonoids are a group of natural poly-phenolic compounds found in plants and have a variety of biological effects and play important role in detoxification of free radicals [30]. Rutin is flavonoid glycosides that are present in herbs and plant foods and possessed different protective effects in vitro as well as in vivo [31, 32] against lipid peroxidation and oxidative stress-mediated diseases [33]. Rutin is an immuno-modulator and has anti-oxidant, anti-diarrheal, anti-tumor, and anti-inflammatory effect, myocardial protection, and has renal protective effects against the ischemia-reperfusion-induced renal injury [34]. Therefore, this study investigated the possible protective effects of rutin against cisplatin-induced nephrotoxicity in rats.

## Methods

### Animals

The study was approved by the Research Ethics Committee of the College of Pharmacy, King Saud University. Male Wistar rats (230–260 g) were obtained from College of Pharmacy, King Saud University Animal Care Center and were kept under standard conditions of temperature (22 ± 1 °C), humidity (50–55%), and a 12-h light:/dark cycle. Food and water were freely available. All methods were conducted in accordance with the Guide for Care and Use of Laboratory Animals, Institute for Laboratory Animal Research, National Institute of Health (NIH publication No. 80–23; 1996).

### Chemicals

Cisplatin (1 mg/ml sterile concentrate) was a gift from King Khalid University Hospital drug store, KSU, KSA. Rutin (CAS Number 207671-50-9) was purchased from Sigma Chemicals (Sigma-Aldrich Louis, MO, USA). Primers were designed using primer express 3 software (Applied Biosystem, Life Technologies, Grand Island, NY, USA) and Syber Green master mix kit (Cat#4309155) were purchased from Applied Biosystems (Life Technologies, Grand Island, NY, USA).

### Experimental design

The experimental Design follows Kamel et al., [3]. The rats were randomly divided into four groups (ten rats each) as follows: Group-I: intraperitoneal (i.p.) received saline (2.5 ml/kg) (normal control group). Group-II: i.p. received single dose 5 mg/kg cisplatin, (cisplatin group) [35]. Group-III: orally received 30 mg/kg rutin dissolved in water for 14 days (Rutin group) [36]. Group-IV: orally received 30 mg/kg rutin, dissolved in water for 14 days with a single dose of cisplatin (5 mg/kg, i.p.) on the tenth day.

All animals were weighted and were exposed to ether and were killed by decapitation 24 h after the last treatment. Blood samples were obtained and sera were separated. The kidney was immediately removed then washed with ice-cold saline solution. Parts of both kidneys were cut into small pieces for histopathological study and for the gene expression analysis.

### Bioassays

#### Determination of blood urea nitrogen and serum creatinine

Blood urea nitrogen (BUN) was measured spectrophotometrically according to the methods of Tobacco et al. [37]. In brief, serum was diluted 1:4 in normal saline and 5 μL of diluted serum and standard (in duplicate) were added to the microplate wells; then 150 μL of urease Mix solution was added to each well. The plate was incubated for 15 min under shaking at room temperature. Then 150 μL of Alkaline Hypochlorite was added to each well. After 10 min’ incubation at room temperature. Measure the absorbance of each sample in duplicate at 620 nm using microplate reader. The blood urea nitrogen concentration was calculated from stander curve. Serum creatinine was measured according to the methods of Fabiny and Ertingshausen [38] in brief, 100 μl of serum samples and standard was mixed with picric acid (17.5 mmol/l final concentration)/sodium hydroxide solution (0.16 mol/l final concentration) after 30 s and 2 min later the absorbance of standard and sample were recorded. After that, the creatinine concertation was calculated by dividing the delta absorbance of the sample by delta absorbance of the control multiply by standard concentration.

### Histopathology examination

The kidneys harvested from each groups were fixed in 10% neutral buffered formaldehyde. Tissues dehydration, clearing in xylene and paraffin embedding was done according to the standard method. Sections were cut by a rotary microtome at 5–7 μm thick, and were stained by haematoxylin and eosin and periodic acid schief (PAS). Sections were examined under a light microscope and findings documented by two certified histopathologists.

### Estimation of Malondialdehyde of lipid peroxidation

Malondialdehyde (MDA) concentration in tissues was measured as it is the major product of membrane lipid peroxidation as a previously described method by Ohkawa et al., [39]. The principle of this method depends on the formation of pink color resulted in reaction between MDA and thiobarbituric acid. This reaction producing a thiobarbituric acid reactive substance (TBARS), pink color, measured spectrophotometrically at 532 nm.

### Estimation of (glutathione) GSH levels in kidney tissues

Glutathione concentration in 200 g kidney tissues homogenate was determined as previously described method by Sedlak and Lindsay [40].

### RNA extraction and Gene expression studies

Total RNAs were extracted from kidneys tissue by Trizol method according to the manufacturer’s protocol as previously described [41]. The quantity was characterized using a UV spectrophotometer. The isolated RNA has an A 260/280 ratio of 1.9–2.1.

### cDNA synthesis and real-time PCR methods

One microgram of total RNA was used to generate cDNA using a SuperScript™ first-strand synthesis system kit (Invitrogen, CA, USA), according to the manufacturer’s instructions. Real-time PCR was done using 2^-ΔΔC*t*^ method according to our previous study [42] and *GAPDH* gene was used as internal control. All primers used in this study were synthesized in Jena Bioscience Germany and were listed in Table 1.

**Table 1:** Primers used in this study

Gene Name	Forward primer	Reverse primer
*JNK*	5′-AAATAGAGCATCCCAGTCTTCGA-3′	5′-ACTGGGCCGCTGTTTCTG-3′
*MKK4*	5′- CATCGGGCCTCCAGCTT -3′	5′- AAATTCAACTTCAGGGCTTTGC -3′
*MKK7*	5′- AAGCTCTGTGACTTTGGCATCA -3′	5′- CAGCCAGCACTCCGTGTTT -3′
*P38*	5′-GGTTTTGGACTCGGATAAGAGGAT-3’	5′-GGGTCGTGGTACTGAGCAAAG-3’
*TRAF2*	5′-ACGCTGCCCGCAGAGA-3’	5′-TCTTTCAAGGTCCCCTTCCA-3’
*TNF-α*	5′-CGGGCTCAGAATTTCCAACA-3’	5′-CGCAATCCAGGCCACTACTT-3’
*IL-1-α*	5′-CATCCGTGGAGCTCTCTTTACA-3’	5′-TTAAATGAACGAAGTGAACAGTACAGATT-3’
*GAPDH*	5′-AACTCCCATTCCTCCACCTT-3’	5′-GAGGGCCTCTCTCTTGCTCT-3’

### Statistical analysis

The data were analyzed using GraphPad Prism 5 (GraphPad Software, Inc., La Jolla, CA, USA). Statistical significance was evaluated by one-way analysis of variance (ANOVA) followed by the Tukey-Kramer multiple comparison tests. All data were expressed as mean ± SEM, *n* = 10. The value of *P* < 0.05 was considered statistically significant.

## Results

### Effects of CP on renal cells

**Fig. 1:**
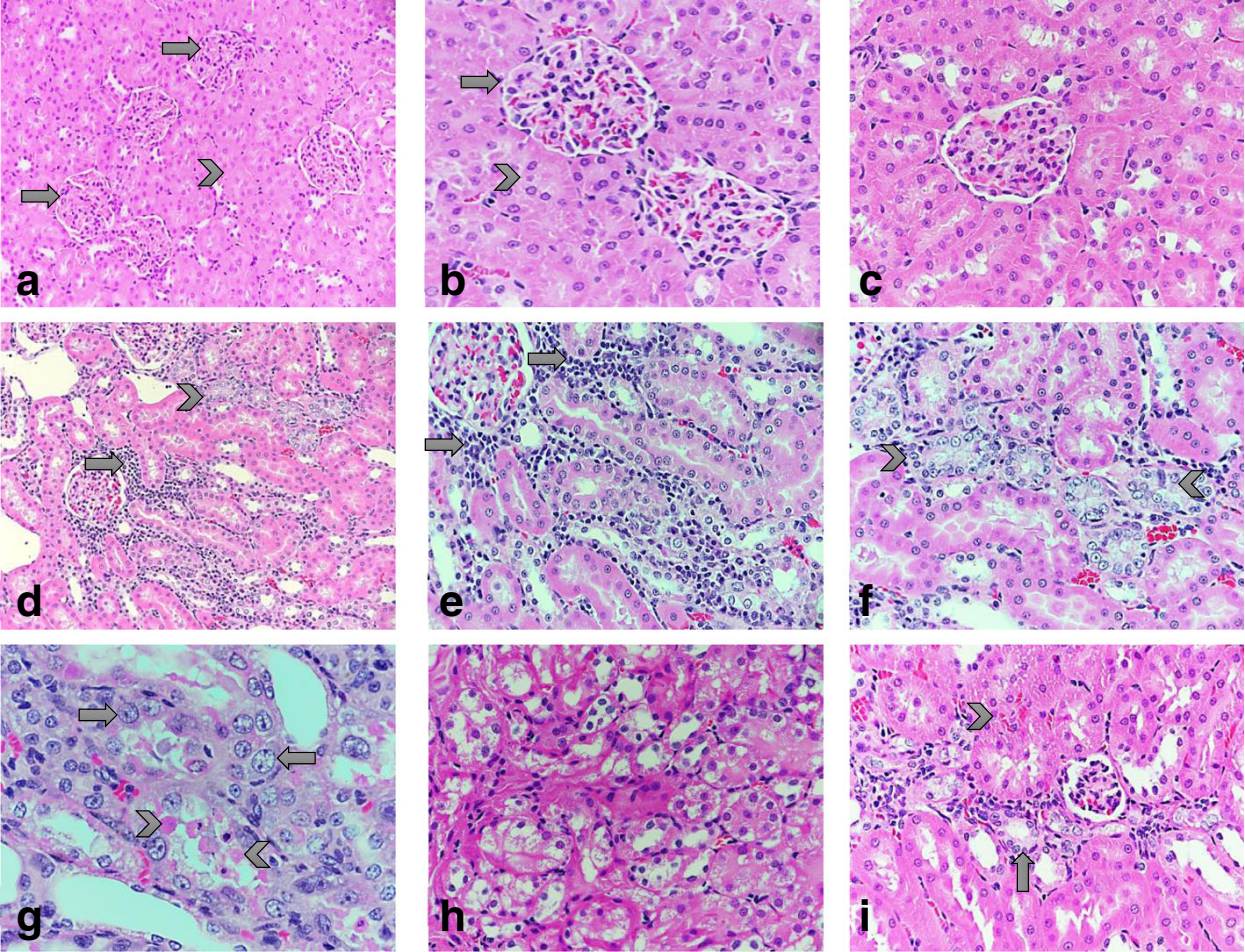
Histological changes in renal tissues in response to cisplatin, rutin, and cisplatin plus rutin: **a** and **b**: photomicrographs of control rat kidney shows normal looking glomeruli (*arrows*) and the tubules (*arrowheads*) with no histological abnormality (hematoxylin-eosin stain, original magnification: ×200 and ×400 respectively). **c**: photomicrograph of rat kidney treated with rutin also show no significant pathological changes. **d**, **e** and **f**: photomicrographs of rat kidney treated with Cisplatin shows patchy lymphoplasmacytic mononuclear chronic inflammatory cell infiltrate in the interstitium (arrows) and patchy mild acute tubular epithelial cell injury (*arrowheads*) and scattered congested interstitial capillaries (hematoxylin-eosin stain, original magnifications: ×200, ×400 and ×400 respectively). **g** and **h**: more photomicrographs of rat kidney treated with Cisplatin showing reactive/reparative atypia of the injured tubular epithelial tubular cell in the form of enlarged nuclei and prominent nucleoli (*arrow*), many apoptotic tubular epithelial cells (*arrowheads*) along with focal cytoplasmic vacuolization the tubular epithelial cells (asterisk) (hematoxylin-eosin stain, original magnifications: ×400 and ×400 respectively). **i**: photomicrographs of rat kidney treated with Cisplatin and Rutin combination shows minimal focal tubular injury of few tubules (*arrows*) and minimal congestion of the interstitial capillaries (arrowhead) (hematoxylin-eosin stain, original magnification: ×400)

The effects of CP and rutin on histological changes in kidney tissues are shown in Fig. 1. The harvested kidneys from the control and treated rat kidneys were studied under a light microscope. The four compartment in the kidney, namely, glomeruli, tubules, interstitium and blood vessels were examined for any histopathological findings. The kidney in the control rats (GI) showed no histopathological abnormality in the glomeruli, tubules, interstitium and blood vessels (Fig. 1a and b). Rats treated with rutin dissolved in water (GII) also showed normal histology with no histopathological findings (Fig. 1c). The kidneys treated with Cisplatin showed histopathological abnormality in the interstitium and the tubules infiltrate (Fig. 1d to h). The interstitium showed patchy mild chronic mononuclear lymphoplasmacytic inflammatory cell infiltrate and mild congestion. The tubules showed patchy acute tubular injury with reactive/ reparative atypia of the tubular epithelial cells. Some tubular epithelial cells also showed cytoplasmic vacuolization and apoptosis. The glomeruli and the blood vessels were not affected. Rats treat with Rutin and Cisplatin combination showed only minimal histopathological findings in the form of minimal interstitial congestion and minimal tubular injury in a few tubules. The glomeruli and the blood vessels appeared normal.

### Effect of CP on the body weight of rats

**Fig. 2:**
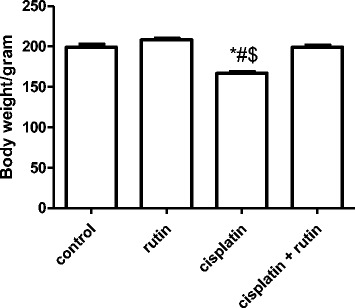
Represent the effect of CP alone, Rutin alone and their combination on the rat body weight. Data were presented as mean ± SEM (*n* = 10). * indicate significant change from control, # indicate significant changes from rutin and $ indicate a significant change Cisplatin plus Rutin

Figure 2 showed the effect of Cisplatin, rutin and their combination on the rat body weight. At the end of the experiment, CP-treated animals significantly lost weight compared to control group (*P < 0.05*). However, administration of rutin alone resulted in an increase in the body weight compared to both control and cisplatin group. Interestingly, administration of rutin in combination with CP resulted in a significant increase the body weight compared to CP group (*p < 0.05*).

### Effects of CP on renal blood urea nitrogen and serum creatinine

**Fig. 3:**
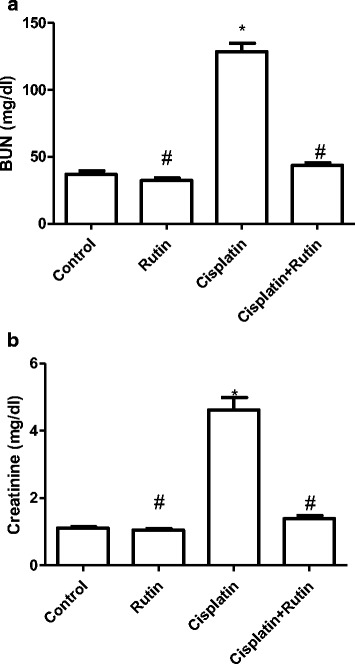
Represents the changes in the levels of serum BUN (**a**) and creatinine (**b**) in rats. Data were presented as mean ± SEM (*n* = 10). * indicate significant change from control, # indicate significant changes from rutin

Blood urea nitrogen (Fig. 3a) and serum creatinine (Fig. 3b) were used as biochemical markers for the nephrotoxicity. CP significant increased the levels of BUN (128.6 ± 44 mg/dl) and serum creatinine (4.6 ± 0.34 mg/dl), compared to control group 37 ± 2.4 mg/dl and 1.2 ± 0.1 mg/dl respectively *(p < 0.001*). The rutin group showed no significant changes in BUN and serum creatinine compared to control group. However, administration of rutin in combination with CP resulted in complete reversal of CP-induced increase in BUN and serum creatinine to their normal values as in control group.

### Assessment of renal oxidative stress

Oxidative stress-induced free radicals that reacted with membrane phospholipids resulted in lipid peroxidation. To investigate the effect of CP, rutin and their combination on the lipid peroxidation biomarkers the MDA level was measured. Cisplatin significantly increased MDA levels in kidney tissue by 230 ± 5.5 nmol/g compared to 68 ± 2.1 nmol/g in control group (*P < 0.001*). Interestingly, the administration of rutin before cisplatin resulted in a reversal of MDA level induced by CP to its normal values as in control group. Administration of rutin alone showed non-significant changes in MDA levels (70 ± 1.8 nmol/g) compared to control group. Also, the free radicals depleted the antioxidant defense GSH. Rats treated with CP had a significant decrease in GSH level by 25 ± 6.8 nmol/100 mg tissue compared to 102 ± 3.5 nmol/100 mg tissue in control group. On the other hand, the administration of rutin before CP lead to increase in the GSH levels from 25 ± 6.8 nmol/100 mg tissue in CP group to 120 ± 3.6 nmol/100 mg tissue (*p < 0.05*). Administration of rutin alone showed non-significant changes in MDA levels 107 ± 2.3 nmol/100 mg tissue compared to the control group.

### The effect of CP on the gene expression levels

**Fig. 4:**
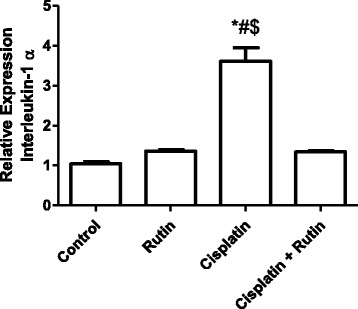
Represents the changes in the expression level of *IL-1α* in Rat kidney tissues induced by CP. Data were presented as mean ± SEM (*n* = 10). * indicate significant change from control, # indicate significant changes from rutin and $ indicate a significant change Cisplatin plus Rutin

To investigate the effect of CP on oxidative stress genes expression levels of *IL-1α* was measured in kidney tissues by using real-time PCR (Fig. 4). CP alone was significantly increased the expression level of *IL-1α* in kidney tissues by 260% (*P < 0.05*) and 164% (*P < 0.001*) compared to control and rutin groups respectively. Interestingly, administration of rutin to CP-treated rats resulted in a complete reversal the reduction of *IL-1α* expression level induced by CP to control values. This reversal change was resulted in significant decrease in *IL-α* expression level by 63% (*p < 0.007*) compared to CP group and by 73% compared to control group.

**Fig. 5:**
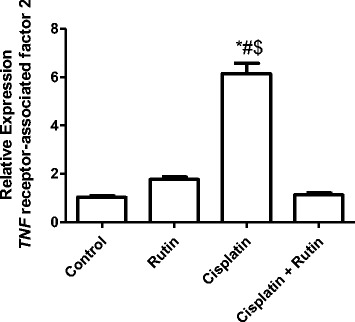
Represents the changes in the expression level of *TRAF2* in Rat kidney tissues induced by CP. Data were presented as mean ± SEM (*n* = 10). * indicate significant change from control, # indicate significant changes from rutin and $ indicate a significant change Cisplatin plus Rutin

Figure 5 showed the effect of CP, rutin and their combination on *TRAF2* expression level in Rat kidney tissues. Cisplatin alone significantly increased the expression level of *TRAF2* in kidney tissues by 491% compared to control group (*P < 0.001*). However, administration of rutin alone resulted in insignificant increase in *TRAF2* expression level by 77% compared to control groups (*P > 0.5*). Interestingly, administration of rutin in combination with CP resulted in significant decrease in the expression level of *TRAF2* compared to CP group (*p < 0.002*).

**Fig. 6:**
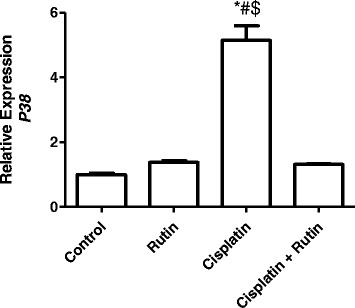
Represents the changes in the expression level of *P38* in Rat kidney tissues induced by CP. Data were presented as mean ± SEM (*n* = 10). * indicate significant change from control, # indicate significant changes from rutin and $ indicate a significant change Cisplatin plus Rutin

Figure 6 showed the effect of CP, Rutin and their combination on the expression levels of *P38* in rat kidney tissues. Treatment of CP alone resulted in significant increase in the *P38* expression level by 410% (*P < 0.001*) in kidney tissues compared to control group. Administration of rutin alone resulted in insignificant increase in the *P38* expression levels by 38% compared to control group (*P < 0.5*). Administration of rutin in combination with CP resulted in significant decrease in the P38 expression level compared to both control and CP groups (*P < 0.001*).

**Fig. 7:**
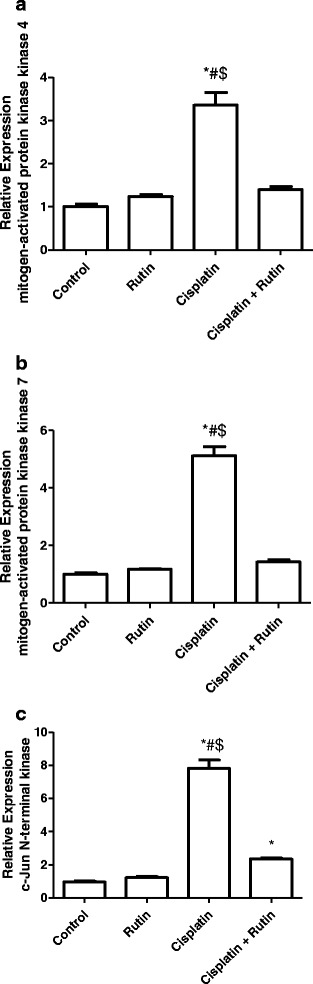
Represents the changes in the expression level of *MKK4* (**a**), *MKK7* (**b**) and *JNK* (**c**) in Rat kidney tissues. Data were presented as mean ± SEM (*n* = 10). * indicate significant change from control, # indicate significant changes from rutin and $ indicate a significant change Cisplatin plus Rutin

Figure 7 showed the effect of CP, Rutin and their combination on the expression level of *MKK4* (A), *MKK7* (B) and *JNK* (C) in kidney tissues. CP alone resulted in significant increase in MKK4 expression level by 236% (*P < 0.001*) in *MKK7* by 412% and in *JNK* by 680% *(P < 0.001*) compared to control group. Interestingly, rutin administration in combination with CP resulted in complete reversal of CP-induced increasing in the expression levels of both *MKK4* and *MKK7* to their normal levels as in control group. On the other hand rutin administration in combination with CP resulted in significant decrease in the expression levels of *JNK* by 71% compared to CP group. There were no significant changes observed in both *MKK4* and *MKK7* in rutin group.

**Fig. 8:**
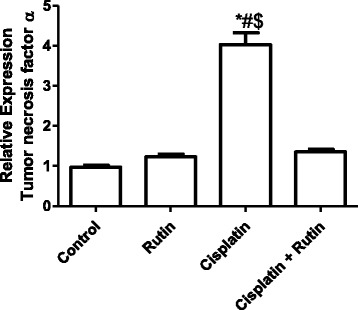
Represents the changes in the expression level of *TNF-α* in Rat kidney tissues. Data were presented as mean ± SEM (*n* = 10). * indicate significant change from control, # indicate significant changes from rutin and $ indicate a significant change Cisplatin plus Rutin

Figure 8 showed the effect of CP, Rutin and their combination on the expression level of *TNF-α* in kidney tissues. CP alone was resulted in significant increase in *TNF-α* expression level by 300% (*P < 0.001*) compared to control group. Interestingly, rutin administration in combination with CP was resulted in complete reversal of CP-induced increasing in the *TNF-α* expression levels to its normal levels as in control group. There were no significant changes observed in *TNF-α* in rutin group.

## Discussion

Cisplatin is an anticancer drug used in the treatment of many types of cancer such as head and neck, lung, testis, ovary, and breast cancers [1, 2]. Nephrotoxicity is the dose-limiting side effect of cisplatin [43] such as acute kidney injury was found in about 20–30% of patients receiving CP [44], Hypo-magnesemia in about 40–100% of patients [45], Fanconi-like syndrome, distal renal tubular acidosis, hypo-calcemia, renal salt wasting and hyper-uricemia [46].

Nephrotoxicity induced by CP is characterized by a reduction in renal function that leads to increasing in serum creatinine and blood urea levels [47]. In the current study, creatinine and BUN serum levels were significantly high in CP-treated rats compared to untreated rats suggesting that CP produced nephrotoxicity as evidenced by the glomerular filtration rate reduction. The elevated serum creatinine and BUN levels induced by CP were significantly restored to their normal levels as in control group by rutin. The rutin protective effect against nephrotoxicity can be attributed to its antioxidant and anti-inflammatory effect on ROS and some cytokines may be involved in the glomerular filtration rate damage [48]. Although the accurate mechanism of CP-induced nephrotoxicity is not well understood, previous study suggested that cisplatin interacts with DNA, through the formation of covalent adducts between certain DNA bases and the platinum compound leading to cell cytotoxicity [49]. Other studies suggest that CP-induced ROS and immune response which are mediators of nephrotoxicity [50–52]. In the present study, the MDA and GSH were measured as biomarkers for the oxidative stress. In the kidney tissue, the MDA level was significantly increased and GSH level was decreased by the effect of cisplatin. However, rutin administration caused significant decreases in lipid peroxidation and promoted increases in GSH content in the kidney. Therefore, rutin can protect the kidney from CP-induced injury via improvement in oxidant status. A similar study found that rutin pre-treatment attenuates renal inflammation and apoptosis induced by cisplatin through reducing TNF-α, NF_k_B and caspase-3 levels [18, 25].

The p38-MAPK stress pathway, stimulated with inflammatory cytokines such as TNF-α or IL-1, act as a key regulator of apoptosis in cells [53]. The expression of the number of inflammatory cytokines and chemokines is increased in the kidney after cisplatin injury [54]. In the present study, CP increased the expression levels of both *TNF-α* or *IL1-α*. Similarly, other study found that the single injection of cisplatin in mice induced nephrotoxicity. In the kidneys of cisplatin-treated mice, the nephrotoxicity caused up-regulation in *TNF-α, IL-1β,* macrophage inflammatory protein-2 (*MIP-2*), monocyte chemoattractant protein-1 (*MCP-1*), *ICAM-1*, and *TGF-β* [55].

The present study showed that the rutin supplementation improved the CP-induced increased in the expression levels of *IL-1* and *TNF-α* that were in agreement with previous reports. Rutin acts as antioxidant and anti-inflammatory and improves renal abnormality induced by several factors or chemotherapeutic agents like doxorubicin or cisplatin [56–58]. *TNF-α* induced by cisplatin is highly dependent upon the production of ROS, activation of *NFκB*, and *p38 MAPK*. However, the activation of *TNF-α* and *IL-1* are involved in several signal transduction mechanisms, including the *NF*_*K*_ *B* and AP-1 pathways. In fact, the stress-activated group of MAPKs (*JNK* and *p38*) is strongly activated by *TNF-α* and *IL-1* [54]. This was in agreement with the present study in which single dose of CP increase the expression levels of *JNK* and *P38*. The activation of *JNK* by *TNF-α* mediated by the TNF receptor-associated factor (*TRAF*) group of adapter proteins [59].

In the current study, the overexpression of *TRAF2* as a result of cisplatin may be the cause of nephrotoxicity and apoptosis. The decrease in the expression level of *TRAF2* in kidney tissues after rutin supplementation in CP-treated rats suggests that rutin may protect against CP-induced nephrotoxicity by regulating apoptotic pathways. Activation of TNF receptors leads to recruitment of the *TRAF2* adapter protein [60, 61]. The activation of the *TRAF2* expression is required for *JNK* activation by *TNF* [62]. A study showed that in nephrotoxicity induced by chemotherapy, genes for *JNK* play an essential role in modulating the pro- and anti-apoptotic proteins located in the mitochondria [63]. *JNK* with ROS can promote apoptosis by inhibiting anti-apoptotic proteins [64]. Also, *JNK* can be activated through its phosphorylation by *MKK4* and *MKK7* at threonine, tyrosine. *MAPKKK* activate both *MKK4* and *MKK7* protein kinases by dual phosphorylation at two sites in the T-loop [65]. The *MKK*7 protein kinase is primarily activated by cytokines (e.g *TNF-α* and *IL-1*) and *MKK4* is primarily activated by environmental stress [66]. In the current study CP- induced the expression levels of *MKK4* and *MKK7* and these alterations attenuated by rutin supplementation in CP-treated rats. P38 MAPK is activated by *MKK3, MKK4* and *MKK6* [67]. In the present study, *P38* expression levels were increased after a single dose of cisplatin. Similarly, several studies suggested that the inhibition of *p38 MAPK*, *ERK* or *JNK* with specific pharmacologic or genetic inhibitors reduced inflammation and renal injury [17, 68, 69].

Rutin administration in CP-treated rat restored the expression levels of *P38* and reduced the apoptosis. Therefore, cisplatin-induced nephrotoxicity can be ameliorated by free radical scavengers [70], iron chelators [71], superoxide dismutase [48] and Vitamin E [72].

## Conclusions

In conclusion, single dose of cisplatin-induced nephrotoxicity through the activation of *P38 MAPK* pathway. Our data may help in understanding the molecular mechanisms of rutin in CP nephrotoxicity. Rutin attenuates CP nephrotoxicity might be through its antioxidant as well as *p38-MAPK* inhibitor properties.

## Abbreviations

*AP-1*: Activator protein 1
BUN: Blood urea nitrogen
CDNA: Complementary deoxynucleic acid
CP: Cisplatin
*GAPDH*: Glyceraldehyde-3-phosphate dehydrogenase
GSH: Glutathione
*IL-1-α*: interleukin-1 alpha
*JNK*: c-Jun N-terminal kinases
*MAPK*: Mitogen-activated protein kinase
*MCP-1*: Monocyte Chemoattractant protein-1
MDA: Malondialdehyde
*MIP2*: Macrophage inflammatory protein-2
*MKK4*: Mitogen-activated protein kinase 4
*MKK7*: Mitogen-activated protein kinase 7
*NF*_*k*_*B*: Nuclear factor kappa beta
*P38*: P38 mitogen-activated protein kinases
*RANTES*: Regulated on Activation Normal T Expressed and Secreted
RNA: Ribonucleic acid
RNS: Reactive Nitrogen Species
ROS: Reactive oxygen species
TBARS: Thiobarbituric acid reactive substance
*TGF-β*: Transforming growth factor-beta
*TNF-α*: tumor necrosis factors alpha
*TRAF2*: TNF Receptor-Associated Factor 2
UV: Ultraviolet

## References

[1] Badary OA, Abdel-Maksoud S, Ahmed WA, Owieda GH (2005). Naringenin attenuates cisplatin nephrotoxicity in rats. Life Sci.

[2] Rabik CA, Dolan ME (2007). Molecular mechanisms of resistance and toxicity associated with platinating agents. Cancer Treat rev.

[3] Kamel KM, Abd El-Raouf OM, Metwally SA, Abd El-Latif HA, El-sayed ME (2014). Hesperidin and rutin, antioxidant citrus flavonoids, attenuate cisplatin-induced nephrotoxicity in rats. J Biochem Mol Toxicol.

[4] Li H, Tang Y, Wen L, Kong X, Chen X, Liu P, Zhou Z, Chen W, Xiao C, Xiao P (2017). Neferine reduces cisplatin-induced nephrotoxicity by enhancing autophagy via the AMPK/mTOR signaling pathway. Biochem Biophys res Commun.

[5] Lebwohl D, Canetta R (1998). Clinical development of platinum complexes in cancer therapy: an historical perspective and an update. Eur J Cancer.

[6] Zhu X, Jiang X, Li A, Zhao Z, Li S. S-Allylmercaptocysteine attenuates Cisplatin-induced Nephrotoxicity through suppression of apoptosis, oxidative stress, and inflammation. Nutrients. 2017:9(2).

[7] Luke DR, Vadiei K, Lopez-Berestein G (1992). Role of vascular congestion in cisplatin-induced acute renal failure in the rat. Nephrol Dial Transplant.

[8] Kumar P, Sulakhiya K, Barua CC, Mundhe N. TNF-alpha, IL-6 and IL-10 expressions, responsible for disparity in action of curcumin against cisplatin-induced nephrotoxicity in rats. Mol Cell Biochem. 2017; 10.1007/s11010-017-2981-5 28258441

[9] Chtourou Y, Aouey B, Kebieche M, Fetoui H (2015). Protective role of naringin against cisplatin induced oxidative stress, inflammatory response and apoptosis in rat striatum via suppressing ROS-mediated NF-kappaB and P53 signaling pathways. Chem Biol Interact.

[10] Lin L, Zheng J, Zhu W, Jia N (2015). Nephroprotective effect of gelsemine against cisplatin-induced toxicity is mediated via attenuation of oxidative stress. Cell Biochem Biophys.

[11] Oh CJ, Ha CM, Choi YK, Park S, Choe MS, Jeoung NH, et al. Pyruvate dehydrogenase kinase 4 deficiency attenuates cisplatin-induced acute kidney injury. Kidney Int. 2017;91(4):880–95. 10.1016/j.kint.2016.10.011 28040265

[12] Saral S, Ozcelik E, Cetin A, Saral O, Basak N, Aydin M, Ciftci O (2016). Protective role of *Diospyros lotus* on cisplatin-induced changes in sperm characteristics, testicular damage and oxidative stress in rats. Andrologia.

[13] Pedraza-Chaverri J, Barrera D, Maldonado PD, Chirino YI, Macias-Ruvalcaba NA, Medina-Campos ON, Castro L, Salcedo MI, Hernandez-Pando R (2004). S-allylmercaptocysteine scavenges hydroxyl radical and singlet oxygen in vitro and attenuates gentamicin-induced oxidative and nitrosative stress and renal damage in vivo. BMC Clin Pharmacol.

[14] Divya MK, Lincy L, Raghavamenon AC, Babu TD (2016). Ameliorative effect of Apodytes dimidiata on cisplatin-induced nephrotoxicity in Wistar rats. Pharm Biol.

[15] Romero F, Perez M, Chavez M, Parra G, Durante P (2009). Effect of uric acid on gentamicin-induced nephrotoxicity in rats - role of matrix metalloproteinases 2 and 9. Basic Clin Pharmacol Toxicol.

[16] Malik S, Suchal K, Bhatia J, Khan SI, Vasisth S, Tomar A, Goyal S, Kumar R, Arya DS, Ojha SK (2016). Therapeutic potential and molecular mechanisms of Emblica officinalis Gaertn in countering Nephrotoxicity in rats induced by the chemotherapeutic agent Cisplatin. Front Pharmacol.

[17] Pabla N, Dong Z (2008). Cisplatin nephrotoxicity: mechanisms and renoprotective strategies. Kidney Int.

[18] Abdel-Daim MM, El-Sayed YS, Eldaim MA, Ibrahim A (2017). Nephroprotective efficacy of ceftriaxone against cisplatin-induced subchronic renal fibrosis in rats. Naunyn Schmiedeberg's Arch Pharmacol.

[19] Yamaguchi K, Ishikawa T, Kondo Y, Fujisawa M (2008). Cisplatin regulates Sertoli cell expression of transferrin and interleukins. Mol Cell Endocrinol.

[20] Ma X, Dang C, Kang H, Dai Z, Lin S, Guan H, Liu X, Wang X, Hui W (2015). Saikosaponin-D reduces cisplatin-induced nephrotoxicity by repressing ROS-mediated activation of MAPK and NF-kappaB signalling pathways. Int Immunopharmacol.

[21] Arany I, Megyesi JK, Kaneto H, Price PM, Safirstein RL (2004). Cisplatin-induced cell death is EGFR/src/ERK signaling dependent in mouse proximal tubule cells. Am J Physiol Renal Physiol.

[22] Nowak G (2002). Protein kinase C-alpha and ERK1/2 mediate mitochondrial dysfunction, decreases in active Na+ transport, and cisplatin-induced apoptosis in renal cells. J Biol Chem.

[23] Koul HK, Pal M, Koul S (2013). Role of p38 MAP Kinase signal transduction in solid tumors. Genes Cancer.

[24] Malik S, Suchal K, Gamad N, Dinda AK, Arya DS, Bhatia J (2015). Telmisartan ameliorates cisplatin-induced nephrotoxicity by inhibiting MAPK mediated inflammation and apoptosis. Eur J Pharmacol.

[25] Dunn E, Sims JE, Nicklin MJ, O'Neill LA (2001). Annotating genes with potential roles in the immune system: six new members of the IL-1 family. Trends Immunol.

[26] Gaestel M, Kotlyarov A, Kracht M (2009). Targeting innate immunity protein kinase signalling in inflammation. Nat rev Drug Discov.

[27] Krause A, Holtmann H, Eickemeier S, Winzen R, Szamel M, Resch K, Saklatvala J, Kracht M (1998). Stress-activated protein kinase/Jun N-terminal kinase is required for interleukin (IL)-1-induced IL-6 and IL-8 gene expression in the human epidermal carcinoma cell line KB. J Biol Chem.

[28] Finch A, Holland P, Cooper J, Saklatvala J, Kracht M (1997). Selective activation of JNK/SAPK by interleukin-1 in rabbit liver is mediated by MKK7. FEBS Lett.

[29] Kracht M, Shiroo M, Marshall CJ, Hsuan JJ, Saklatvala J (1994). Interleukin-1 activates a novel protein kinase that phosphorylates the epidermal-growth-factor receptor peptide T669. Biochem J.

[30] AlSharari SD, Al-Rejaie SS, Abuohashish HM, Ahmed MM, Hafez MM (2016). Rutin attenuates Hepatotoxicity in high-cholesterol-diet-fed rats. Oxidative med Cell Longev.

[31] Deschner EE, Ruperto J, Wong G, Newmark HL (1991). Quercetin and rutin as inhibitors of azoxymethanol-induced colonic neoplasia. Carcinogenesis.

[32] Imam F, Al-Harbi NO, Al-Harbia MM, Korashy HM, Ansari MA, Sayed-Ahmed MM, Nagi MN, Iqbal M, Khalid Anwer M, Kazmi I (2017). Rutin attenuates Carfilzomib-induced Cardiotoxicity through inhibition of NF-kappaB, hypertrophic Gene expression and oxidative stress. Cardiovasc Toxicol.

[33] Lopez-Revuelta A, Sanchez-Gallego JI, Hernandez-Hernandez A, Sanchez-Yague J, Llanillo M (2006). Membrane cholesterol contents influence the protective effects of quercetin and rutin in erythrocytes damaged by oxidative stress. Chem Biol Interact.

[34] Herrmann AP, Janke HD (2001). Cofermentation of rutin and hesperidin during two-stage anaerobic pre-treatment of high-loaded brewery wastewater. Water res.

[35] Naghizadeh B, Boroushaki MT, Vahdati Mashhadian N, Mansouri MT (2008). Protective effects of crocin against cisplatin-induced acute renal failure and oxidative stress in rats. Iran Biomed J.

[36] Shimoi K, Shen B, Toyokuni S, Mochizuki R, Furugori M, Kinae N (1997). Protection by alpha G-rutin, a water-soluble antioxidant flavonoid, against renal damage in mice treated with ferric nitrilotriacetate. Jpn J Cancer Res Gann.

[37] Tabacco A, Meiattini F, Moda E, Tarli P (1979). Simplified enzymic/colorimetric serum urea nitrogen determination. Clin Chem.

[38] Fabiny DL, Ertingshausen G (1971). Automated reaction-rate method for determination of serum creatinine with the CentrifiChem. Clin Chem.

[39] Ohkawa H, Ohishi N, Yagi K (1979). Assay for lipid peroxides in animal tissues by thiobarbituric acid reaction. Anal Biochem.

[40] Sedlak J, Lindsay RH (1968). Estimation of total, protein-bound, and nonprotein sulfhydryl groups in tissue with Ellman's reagent. Anal Biochem.

[41] Chomczynski P (1993). A reagent for the single-step simultaneous isolation of RNA, DNA and proteins from cell and tissue samples. BioTechniques.

[42] Sayed-Ahmed MM, Al-Shabanah OA, Hafez MM, Aleisa AM, Al-Rejaie SS (2010). Inhibition of gene expression of heart fatty acid binding protein and organic cation/carnitine transporter in doxorubicin cardiomyopathic rat model. Eur J Pharmacol.

[43] Sastry J, Kellie SJ (2005). Severe neurotoxicity, ototoxicity and nephrotoxicity following high-dose cisplatin and amifostine. Pediatr Hematol Oncol.

[44] Madias NE, Harrington JT (1978). Platinum nephrotoxicity. Am J med.

[45] Schilsky RL, Anderson T (1979). Hypomagnesemia and renal magnesium wasting in patients receiving cisplatin. Ann Intern med.

[46] Peres LA, da Cunha AD, Jr. (2013). Acute nephrotoxicity of cisplatin: molecular mechanisms. J Bras Nefrol.

[47] Farooqui Z, Ahmed F, Rizwan S, Shahid F, Khan AA, Khan F (2017). Protective effect of *Nigella sativa* oil on cisplatin induced nephrotoxicity and oxidative damage in rat kidney. Biomed Pharmacother.

[48] Cuzzocrea S, Mazzon E, Dugo L, Serraino I, Di Paola R, Britti D, De Sarro A, Pierpaoli S, Caputi A, Masini E (2002). A role for superoxide in gentamicin-mediated nephropathy in rats. Eur J Pharmacol.

[49] Ibrahim A, Eldaim MA, Abdel-Daim MM (2016). Nephroprotective effect of bee honey and royal jelly against subchronic cisplatin toxicity in rats. Cytotechnology.

[50] Pan H, Mukhopadhyay P, Rajesh M, Patel V, Mukhopadhyay B, Gao B, Hasko G, Pacher P (2009). Cannabidiol attenuates cisplatin-induced nephrotoxicity by decreasing oxidative/nitrosative stress, inflammation, and cell death. J Pharmacol exp Ther.

[51] Rashed LA, Hashem RM, Soliman HM (2011). Oxytocin inhibits NADPH oxidase and P38 MAPK in cisplatin-induced nephrotoxicity. Biomed Pharmacother.

[52] Abdellatief SA, Galal AA, Farouk SM, Abdel-Daim MM (2017). Ameliorative effect of parsley oil on cisplatin-induced hepato-cardiotoxicity: a biochemical, histopathological, and immunohistochemical study. Biomed Pharmacother.

[53] Suchal K, Malik S, Khan SI, Malhotra RK, Goyal SN, Bhatia J, Kumari S, Ojha S, Arya DS (2017). Protective effect of mangiferin on myocardial ischemia-reperfusion injury in streptozotocin-induced diabetic rats: role of AGE-RAGE/MAPK pathways. Scientific Reports.

[54] Chowdhury S, Sinha K, Banerjee S, Sil PC (2016). Taurine protects cisplatin induced cardiotoxicity by modulating inflammatory and endoplasmic reticulum stress responses. Biofactors.

[55] Ramesh G, Reeves WB (2004). Inflammatory cytokines in acute renal failure. Kidney Int Suppl.

[56] Korkmaz A, Kolankaya D (2010). Protective effect of rutin on the ischemia/reperfusion induced damage in rat kidney. J Surg res.

[57] Al-Rejaie SS, Abuohashish HM, Alkhamees OA, Aleisa AM, Alroujayee AS (2012). Gender difference following high cholesterol diet induced renal injury and the protective role of rutin and ascorbic acid combination in Wistar albino rats. Lipids Health Dis.

[58] Peng CC, Hsieh CL, Ker YB, Wang HY, Chen KC, Peng RY (2012). Selected nutraceutic screening by therapeutic effects on doxorubicin-induced chronic kidney disease. Mol Nutr Food res.

[59] Davis RJ (2000). Signal transduction by the JNK group of MAP kinases. Cell.

[60] Rothe M, Wong SC, Henzel WJ, Goeddel DV (1994). A novel family of putative signal transducers associated with the cytoplasmic domain of the 75 kDa tumor necrosis factor receptor. Cell.

[61] Hashem RM, Mohamed RH, Abo-El-matty DM (2016). Effect of curcumin on TNFR2 and TRAF2 in unilateral ureteral obstruction in rats. Nutrition.

[62] Yeh WC, Shahinian A, Speiser D, Kraunus J, Billia F, Wakeham A, de la Pompa JL, Ferrick D, Hum B, Iscove N (1997). Early lethality, functional NF-kappaB activation, and increased sensitivity to TNF-induced cell death in TRAF2-deficient mice. Immunity.

[63] Uehara T, Yamate J, Torii M, Maruyama T (2011). Comparative nephrotoxicity of Cisplatin and nedaplatin: mechanisms and histopathological characteristics. J Toxicol Pathol.

[64] Weijun Wang HS, Che Y, Jiang X (2016). Rasfonin promotes autophagy and apoptosis via upregulation of reactive oxygen species (ROS)/JNK pathway. An Int J Fungal Biol.

[65] Tournier C, Dong C, Turner TK, Jones SN, Flavell RA, Davis RJ (2001). MKK7 is an essential component of the JNK signal transduction pathway activated by proinflammatory cytokines. Genes dev.

[66] Hommes DW, Peppelenbosch MP, van Deventer SJ (2003). Mitogen activated protein (MAP) kinase signal transduction pathways and novel anti-inflammatory targets. Gut.

[67] Cederbaum AI, Lu Y, Wang X, Wu D (2015). Synergistic toxic interactions between CYP2E1, LPS/TNFalpha, and JNK/p38 MAP kinase and their implications in alcohol-induced liver injury. Adv exp med Biol.

[68] Mahran YF, Khalifa AE, El-Demerdash E (2011). A comparative study of protective mechanisms of glycine and L-arginine against cisplatin-induced nephrotoxicity in rat renal cortical slices. Drug Discoveries Therapeutics.

[69] Hanigan MH, Devarajan P (2003). Cisplatin nephrotoxicity: molecular mechanisms. Cancer Therapy.

[70] Alam J, Subhan F, Ullah I, Shahid M, Ali G, Sewell RD (2017). Synthetic and natural antioxidants attenuate cisplatin-induced vomiting. BMC Pharmacol Toxicol.

[71] Mundhe NA, Kumar P, Ahmed S, Jamdade V, Mundhe S, Lahkar M (2015). Nordihydroguaiaretic acid ameliorates cisplatin induced nephrotoxicity and potentiates its anti-tumor activity in DMBA induced breast cancer in female Sprague-Dawley rats. Int Immunopharmacol.

[72] Villani V, Zucchella C, Cristalli G, Galie E, Bianco F, Giannarelli D, Carpano S, Spriano G, Pace A (2016). Vitamin E neuroprotection against cisplatin ototoxicity: preliminary results from a randomized, placebo-controlled trial. Head Neck.

